# Validation of an In-House ELISA Method in the Diagnosis of Cutaneous Leishmaniasis Caused by *Leishmania donovani* in Hambantota District, Sri Lanka

**DOI:** 10.3390/microorganisms10050921

**Published:** 2022-04-27

**Authors:** Nirmitha Lalindi De Silva, Viraji Nefertiti Hiromel De Silva, Arachchige Theja Hemapala Deerasinghe, Upeksha Lakmini Rathnapala, Hirotomo Kato, Makoto Itoh, Hidekazu Takagi, Mirani Vasanthamala Weerasooriya, Thishan Channa Yahathugoda

**Affiliations:** 1 Department of Parasitology, Faculty of Medicine, University of Ruhuna, Galle 80000, Sri Lanka; miraniweera@yahoo.co.uk (M.V.W.); tcyahath@med.ruh.ac.lk (T.C.Y.); 2 Base Hospital Tangalle, Tangalle 82200, Sri Lanka; virajine@gmail.com; 3 District General Hospital Hambantota, Hambantota 82000, Sri Lanka; athdeerasinghe@yahoo.com; 4 School of Biosciences, University of Melbourne, Melbourne, VIC 3010, Australia; gurathnapala@unimelb.edu.au; 5 Division of Medical Zoology, Department of Infection and Immunity, Jichi Medical University, Tochigi 329-0498, Japan; hirok@jichi.ac.jp; 6 Department of Microbiology and Immunology, Aichi Medical University School of Medicine, Aichi 480-1195, Japan; macfilaria@gmail.com (M.I.); htakagi@aichi-med-u.ac.jp (H.T.)

**Keywords:** cutaneous leishmaniasis, diagnosis, ELISA, rKRP42, rK39, serology, serum, urine, Sri Lanka

## Abstract

Clinical diagnosis has become a challenge amidst a surge of cutaneous leishmaniasis in Southern Sri Lanka. The routine diagnostic method, slit-skin smear (SSS), has variable sensitivity, leading to undiagnosed cases. Improved diagnostics are urgently needed. We assessed a new in-house ELISA method for its diagnostic capabilities against ITS-1 nested PCR (gold standard—Gs). A cohort of 190 clinical CL cases was examined by SSS microscopy, anti-rKRP42 IgG ELISA (serum- and urine-based), and rK39-Immunochromatographic strip test. Validation was done using non-endemic sera, and cutoffs were developed using the receiver operating curve. The sensitivity of SSS for case detection was 77.9% (authors) and 76.3% (technicians). ELISA vs. Gs demonstrated sensitivity (Sn) = 94.4%; specificity (Sp) = 50.0%; positive predictive value (PPV) = 97.1%; negative predictive value (NPV) = 33.3%; Kappa agreement (Kp) = 0.39/*p* < 0.01. Comparison of the combination method (SSS by technicians and ELISA) vs. Gs showed: Sn = 98.9%; Sp = 30.0; PPV = 96.2; NPV 60.0%; Kp = 0.378/*p* < 0.01. All methods performed better compared to SSS (29.4%) where the clinical diagnosis was doubtful (PCR = 94.15%; serum ELISA = 88.2%; combination = 94.1%; *p* < 0.01 for all). High serum anti-rKRP42 titers were seen in those with multiple lesions. Anti-rKRP42 urine ELISA was suboptimal as a diagnostic test. A 9% rate of positivity was seen for rk39-ICT, and positives recorded high anti-rKRP42 titers. The diagnostic accuracy can be increased above the level of the Gs by combining SSS and ELISA. Advanced studies are required to understand the association between rk39-ICT positivity and high anti-rKRP42 titers.

## 1. Introduction

Leishmaniasis is a neglected tropical disease caused by over 20 species of protozoan parasites belonging to the genus *Leishmania* [1]. The disease is transmitted through bites of infected female sandflies in the genera *Phlebotomus* or *Lutzomyia* [1]. Over the past two decades, many new endemic regions and epidemics have been reported globally [2,3,4]. Sri Lanka is such a focus; it was first recognized as endemic for the cutaneous form of the disease in 1992 [5] and there has been an island-wide spread and rise in the caseloads since. The causative parasite was identified as *Leishmania donovani* zymodeme MON-37 of the *L. donovani* complex, a naturally attenuated parasite form that causes visceral leishmaniasis (VL) in India and East Africa and cutaneous leishmaniasis (CL) in Cyprus [2,6]. The probable vector is *Phlebotomus argentipes* (Annandale and Brunette 1908) [7,8]. A few locally acquired VL and mucocutaneous leishmaniasis (MCL) cases were also reported, demonstrating the visceralizing potential of the Sri Lankan strain [9,10]. Although amastigotes and *Leishmania* DNA have been isolated from domestic dogs [11,12], it remains unclear whether dongs act as an animal reservoir in Sri Lanka or whether the transmission is primarily human-to-human. Considering the rapidly escalating disease burden and uncertainty about animal reservoirs, detecting and treating every human case is imperative for control of the transmission.

Diagnosis of CL in Sri Lanka largely relies on clinical diagnosis followed by slit-skin smear microscopy (SSS) which has variable sensitivity, sometimes as low as 33% in local studies [13]. Serology using serum and urine is a well-established and widely used tool in VL [12,13] due to a high humoral response [14,15,16]. Urine has the added advantage of non-invasive collection and community acceptance, making it favorable, especially for population-based studies. In comparison, the humoral response to CL is of lower magnitude rendering antibody-based tests less sensitive for CL. However, the observation that pre- and post-treatment serological responses differ fueled interest in serodiagnosis of CL [17]. Different serological assays have varying sensitivity levels, and some have been promising [18,19]. Recent studies in Sri Lanka have estimated a seroprevalence rate of 34% among CL cases [20]. A follow-up study revealed a higher seroprevalence of 82% using crude protein lysate of the local dermatropic strain [21]. Even though CL in Sri Lanka is caused by *L. donovani*, a normally visceralizing parasite, the rK39 antibody detection has been consistently low in Sri Lanka [20,22].

Recombinant kinesin-related protein-42 (rKRP42) antigen, a subunit of *L. donovani* kinesin-related protein, is a homolog of rK39 (comparable in sequence with 39 extra amino acids) [23]. Urine-based ELISA to detect antibodies against rKRP42 antigen has been found to have comparable sensitivity to serum-based ELISA (94.6%) in patients with VL caused by *L. donovani* [24], suggesting urine rKRP42 ELISA could predict clinical cases and, thereby, possible outbreaks. The possibility of large-scale production of recombinant antigens offers an added advantage compared to crude antigen lysates which require propagation of parasites in culture and preparation. Serology offers supportive diagnostic evidence by less invasive means than invasive tissue sampling, albeit the common limitations of serology in an endemic setting. The present study evaluated the seroprevalence and diagnostic ability of rKRP42 IgG-based ELISA among CL caused by *L. donovani* in the Hambantota district of southern Sri Lanka, which is one of the districts heavily impacted by CL [25].

## 2. Materials and Methods

### 2.1. Study Area

The study was conducted in the Hambantota District in southern Sri Lanka. The land area is generally a flat lowland, and much of the area is dry savanna. There are several stretches of rice fields. Coconut plantations are few and mainly confined to home gardens.

### 2.2. Sample Collection

#### 2.2.1. Patients with Cutaneous Leishmaniasis (CL)

We recruited residents of the Hambantota District, aged 18 years or more, presenting for the first time to the dermatology clinic of the selected study site (Base Hospital Tangalle) with skin lesions suggestive of CL. Based on physical examination the dermatology specialist classified the lesions as either clinically confirmed or doubtful. Slit-skin smears and a 3 mm punch biopsy were collected from the lesions. Diagnoses were considered confirmed if amastigotes were seen on the Giemsa stained slit-skin smear (SSS) or *Leishmania* DNA detected by nested ITS-1 PCR. Two SSS were prepared from each lesion and were independently examined by the study authors and the technicians of the hospital laboratory. Venous blood (2 mL) and urine (5 mL) were collected from each participant. Blood was transported to the laboratory on ice, allowed to clot at room temperature for one hour, and centrifuged at 2500 rpm for 10 min. Separated serum was stored at −20 °C until analyzed. Urine was preserved using sodium azide at 0.1%, transported at room temperature, and stored at 4 °C until analysis. Demographic data, details of the clinical presentation, and risk factors predisposing to CL were recorded.

#### 2.2.2. Control Groups

The study enrolled two control groups, the endemic control (EC) and Japanese control (JC) groups. For the EC, urine was collected from 255 age and gender-matched subjects with no clinical evidence of CL. EC blood samples were not available for this study. Since true non-endemic healthy control samples from Sri Lanka could not be obtained (because all districts are considered endemic at present), serum (*n* = 80) and urine samples (*n* = 80) of healthy individuals from a reference laboratory in Japan (JC) were used to determine assay cutoff values.

### 2.3. Molecular Diagnosis

Nested ITS-1 PCR was performed with DNA extracted from punch biopsy specimens using the QIAGEN DNeasy® (Thermo Fisher Scientific, MA, USA) blood and tissue kit. ITS region amplification was performed using outer primers—LITSR: 5′-CTGGATCATTTTCCGATG-3′ and L5.8S: 5′-TGATACCACTTATCGCACTT-3′ [26] and two novel inner primers—LITSR-inner: 5′-CATTTTCCGATGATTACACC-3′ and L5.8S-inner: 5′-TACTGCGTTCTTCAACGA-3′ (De Silva et al., 2022 manuscript in preparation). The inner primers were designed based on the conserved sequences among several species, viz. *L. donovani, L. infantum, L. major, L. tropica*, and *L. aethiopica*. Nested ITS1 PCR was used as the gold/reference standard for the present study.

### 2.4. ELISA to Detect Anti-rKRP42 IgG in Serum and Urine

We used the recombinant KRP42 IgG ELISA protocol of Islam et al. [23,24] with minor modifications. Both serum and urine samples were tested for the CL and JC cohorts. In an earlier study, serum assay was optimized for VL sera at 1/4000 dilution [23]. During the optimization process, serum was tested at 1/1000 and 1/4000 while urine was tested undiluted and at ½ dilution. Serum and urine samples were tested in duplicate; serum samples were diluted at 1:4000 and urine at 1:2, which were identified as optimum dilutions, using casein buffer.

Flat-bottomed 96-well microtiter plates (NUNC^TM^, Thermo Fisher Scientific, MA, USA) were coated with 100 µL/well rKRP42 antigen (1 µg/mL) and incubated overnight at 4 °C. The wells were then washed three times with PBS-Tween, pH 7.4 before blocking with casein buffer 200 µL/well (1% casein in 0.05 M Tris-HCl buffer with 0.15 M NaCl, pH 7.6) for 2 h at room temperature (~25 °C). After discarding the blocking buffer, 100 µL of diluted serum/urine samples were added to the wells and incubated overnight at room temperature (~25 °C). Urine and serum were tested on separate plates. After washing the wells three times, a secondary antibody, goat anti-human IgG HRPO conjugate (Invitrogen Corporation, Camarillo, CA, USA), diluted 1:10,000 with casein buffer, was added (100 µL/well) and incubated at 37 °C for 1 h. After washing the wells three times, 2,2′-azino-bis (3-ethylbenzothiazoline-6-sulphonic acid) (ABTS) substrate was added and incubated for 2 h at room temperature. The absorbance of the wells was measured with an ELISA plate reader (BioTek™ ELx808, BioTek® Instruments, Inc., Highland Park, VT, USA) at 405 nm, and the reference was set to 490 nm. Samples were retested if the absorbance value of duplicates differed >40% from their average and if the IgG unit of one well was above the cutoff and the other was below. Antibody levels were arbitrarily expressed as units based on a standard curve. Serial threefold dilutions of pooled positive sera of VL patients were used to construct the standard curve for each plate, and the unit values were estimated from this. Serial dilutions of positive sera, starting from ×20,000 (arbitrary value assigned = 7290 units) and ×180,000 (810 units) were used for serum and urine, respectively. Cutoff values were determined using receiver operating curve (ROC curve) analysis. The cutoff value was 27.5 units (at the sensitivity and specificity of 94.4%) for serum and 6.5 units (at the sensitivity and specificity of 61.7% and 66.8%, respectively) for urine.

### 2.5. Rapid Test (Kalazar Detect™) to Detect Anti-rK39 Antibodies in a Subset of Patients with Confirmed CL

Anti-rKRP42 IgG titers obtained from ELISA among the CL cases (*n* = 190) were classified into quartiles. Twenty-five sera from each quartile were tested with the rK39 rapid test (InBios International, Inc., Seattle, WA, USA).

### 2.6. Data Management and Statistical Analysis

IBM SPSS Statistics Version 25 was used for data analysis. DNA detection by modified nested PCR was employed as the gold standard. Sensitivity (SN), specificity (SP), positive predictive value (PPV), and negative predictive value (NPV) were estimated for each method in comparison to the gold standard. Clinical and demographic data were compared with nested PCR and seropositivity by rKRP42 based serum ELISA by cross-tabulations, and statistical significance was determined by Pearson’s chi-square or Fisher’s exact test. An approximate 2-sided 95% confidence interval (CI) was calculated for each proportion. The Kappa agreement test was performed to analyze the agreement between immunological methods, molecular methods, SSS, and clinical diagnosis. The Landis and Koch scale [27] measured the degree of agreement according to the Kappa value.

### 2.7. Ethical Considerations

Ethical clearance to conduct this study was obtained from the Ethics Review Committee of the Faculty of Medicine, the University of Ruhuna, Sri Lanka (approval no; 19.09.2018:3.5). Informed oral and written consent was taken from all participants before enrollment in the study and sample collection.

## 3. Results

### 3.1. Clinical and Demographic Data

**Figure 1:**
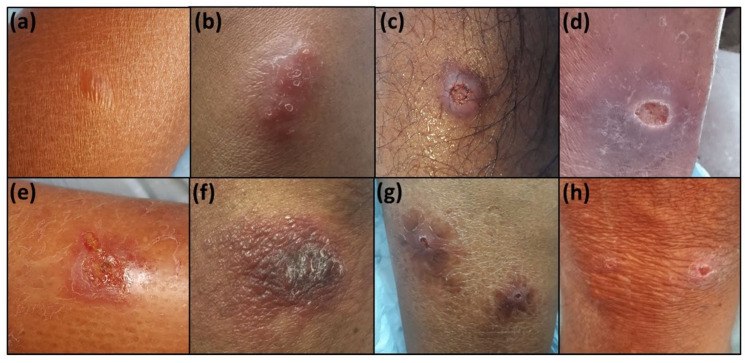
The range of clinical lesion types observed: (**a**) papule; (**b**) nodule; (**c**) ulcerated nodule; (**d**) dry ulcer; (**e**) wet ulcer; (**f**) plaque; (**g**,**h**) multiple lesions.

A total of 190 patients with CL were available for the analysis (Table 1). Among these, 173 were clinically confirmed as CL, whereas 17 patients were doubtful. The male-to-female ratio was 2:1, and age ranged between 19 to 82 years with a mean age of 45.76 (SD ± 13.61). Only 69.5% (132/190) of the participants had an occupation. The majority of the unemployed participants consisted of housewives (48/58; 82.7%). Most participants (88%) belonged to the lower-income categories (LKR<50,000 annually). The majority of cases presented early (<within 4 months of onset (67%) and most lesions were single (80.5%) and small (<2 cm; 86.8%) (Table 1). The mean duration of the lesion at presentation was 4.57 (SD ± 5.94) months. The commonest lesion type was the ulcerated nodules (40%) followed by non-ulcerated nodules (16.8%), dry ulcers (13.7%), papules (12.1%), plaques (10%), and wet ulcers (7.4%) (Figure 1). Over 85% of lesions were on limbs.

Among the control (EC) groups, ages ranged from 18 to 83 years with a mean age of 45.66 (SD ± 14.50). Sixty-three percent were male; age and sex did not differ significantly between the EC and CL groups (*p* = 0.332 and *p* = 0.074 respectively).

### 3.2. Laboratory Diagnosis of CL Patients

#### 3.2.1. Nested PCR Method (Reference Standard of the Study)

One hundred eighty (94.7%) of the clinically diagnosed or predicted CL cases tested positive by nested ITS1 PCR.

#### 3.2.2. Giemsa Stained SSS Microscopy

The hospital technicians rated 76.3% (145/190) of SSS as positive, while the study authors rated 77.9% (148/190) positive. The sensitivity of SSS against nested PCR was 77.8% (technicians).

#### 3.2.3. Serum and Urine Anti-rKRP42 IgG ELISA: Calculation of Cutoff Values and Determination of Sensitivity and Specificity

**Figure 2:**
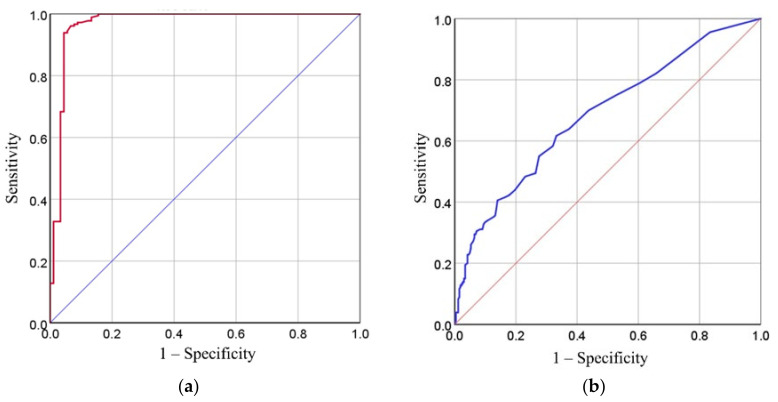
(**a**) ROC curve analysis for rKRP42 serum ELISA; (**b**) ROC curve analysis for anti-rKRP42 urine ELISA.

The cutoff value was 27.5 units (at the sensitivity and specificity of 94.4%) for serum and 6.5 units (at the sensitivity and specificity of 61.7% and 66.8%, respectively) for urine (Figure 2).

#### 3.2.4. Sensitivity, Specificity, PPV, and NPP of Anti-rKRP42 IgG Serum and Urine ELISAs among CL, EC, and JC

**Figure 3:**
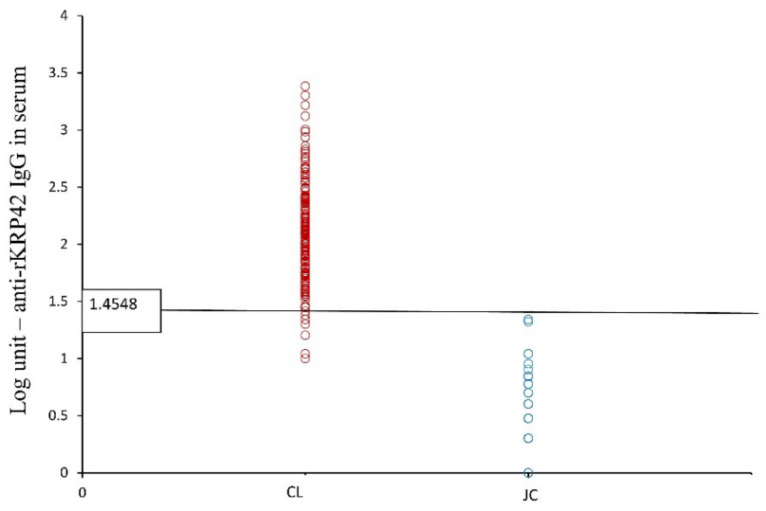
Anti-rKRP42 IgG titers in serum, comparison among CL and Japanese controls (JC).

Serum rKRP42 ELISA detected 170 out of 180 CL cases confirmed by the nested ITS-1 PCR and thereby demonstrated a sensitivity of 94.4% (Table 2). The seropositivity rate within the CL group was significantly higher compared to the JC group, where none were seropositive (0/80, *p* < 0.01) (Table 2; Figure 3). The specificity was 50% and the PPV and NPV were 97.1% and 33.3%, respectively, against the gold standard (nested PCR). Serum ELISA had a fair and significant agreement with the nested PCR (Kappa = 0.360; *p* < 0.01).

**Figure 4:**
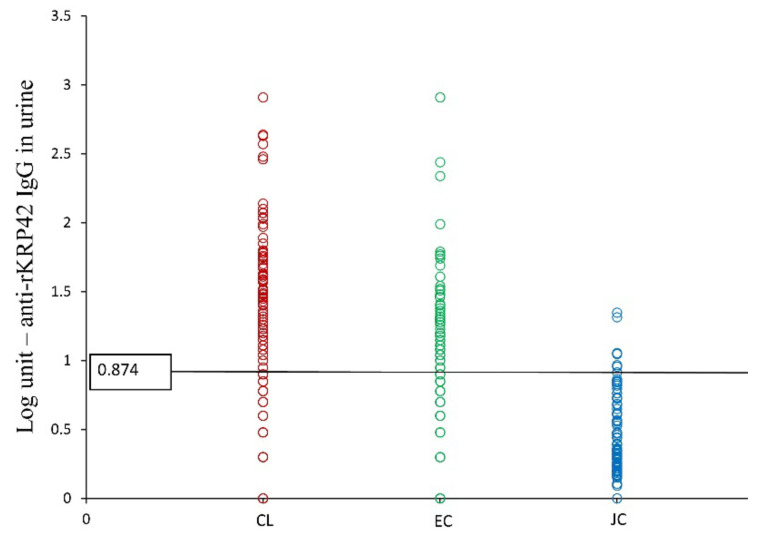
Anti-rKRP42 IgG titers in urine, comparison among groups.

Urine ELISA was positive for only 61.7% of the nested PCR positives; however, the specificity remained 50%, similar to serum ELISA. PPV was high at 95.7%, but NPV was very low (6.8%). Concordance between urine ELISA and nested PCR was poor (Kappa = 0.029, *p* > 0.05). The positive rate by urine ELISA was 32.5% within EC and 3.75% within the JC group (Table 3, Figure 4). However, urine antibody titers were significantly higher (>0.874 Log unit) among the PCR confirmed CL cases compared to all other groups tested (*p* < 0.01) (Figure 4).

### 3.3. Correlation between Serum and Urine Anti-rKRP42 IgG Titers

A fair and significant correlation was observed between IgG titers of serum and urine ELISAs among 190 CL cases (Pearson’s *r* = 0.221; *p* < 0.01).

### 3.4. Combination of SSS and Serum rKRP42 ELISA

If we defined positive cases as having *either* a positive SSS (technician) *or* positive serum ELISA, case detection accuracy improved to 98.9% compared to nested PCR, which had the highest sensitivity as a solitary test in case detection (94.3%) in the present study. Moreover, the combination of SSS and ELISA had a fair and significant agreement with nested PCR (Kappa = 0.378; *p* < 0.01). Specificity was 30% for the combination, but the PPV and NPV were 96.2% and 60%, respectively. The detection rate increased by >20% when the combination is used compared to SSS (technician) used alone to diagnose CL.

### 3.5. The Test Capability in the Diagnosis of Clinically Doubtful Lesions

A comparison was made among the different diagnostic techniques employed for case detection within this study for clinically doubtful lesions (*n* = 17/190) (Table 4). Sixteen of seventeen clinically doubtful lesions tested positive by PCR. The technician rated 5 positive by SSS and the authors 10 positive by SSS. rKRP42 serum ELISA rated 15/17 positive while the combination method rated 16/17 positive. Significance levels were calculated against SSS by technicians, which is the routine diagnostic method. However, PPV and NPP values were calculated against PCR to understand the true diagnostic ability. Diagnostic accuracy of SSS by technicians was very low in clinically doubtful lesions (29.4%); however, the authors managed to detect a higher number of cases (58.8%, *p* = 0.083). All other methods, including nested PCR, rKRP42 serum ELISA, and the combination method, detected a significantly higher number of cases (PCR = 94.15%; serum ELISA = 88.2%; *p* < 0.01 for both). The combination method also detected a significantly higher case number (94.1%; *p* < 0.01) compared to SSS (technicians) alone. The detection rate for the combination method was equal to that of nested PCR (94.1%) (Table 4).

### 3.6. CL Cases Tested by rK39 Rapid Test (Kalazar Detect^™^)

Among the 100 PCR confirmed CL case samples tested, 9% were rK39 test positive. Positivity for rK39 antibodies among the CL cases was significantly associated with high rKRP42 antibody titers. All rK39 positives had rKRP42 titers over the second quartile (unit value >115.6), while 8/9 (88.8%) had rKRP42 antibody titers over the third quartile (unit value >251.3; *p* < 0.01). Overall a higher rate of rK39 antibody positive tests was observed among single (67%; *n* = 6/9; *p* = 0.523), small (88.8%; *n* = 8/9; *p* = 1.000), early lesions of less than 4 months duration (*n* = 8/9; 88.5%; *p* = 0.839), and across all different lesion types except for papules (*n* = 0/9; *p* = 0.275).

### 3.7. Clinico-Demographic Correlations of CL Patients (n = 190) against rKRP42 Serum ELISA

Seroconversion was observed in 175 subjects out of 190. Seroconversion rates compared to nested PCR were slightly higher among males (93.0%) than females (90.7%). Seroconversion was observed among all age categories to an almost equal extent (~92.0%). Although 78.8% (*n* = 138) of seropositive subjects had single skin lesions, seropositivity was significantly higher among people with multiple lesions (100%; *p* < 0.05). Higher antibody titers (anti-rKRP42 IgG >3^rd^ quartile) were observed among people with three or more lesions (*p* < 0.05), and 83.7% (*n* = 31/37) of people with multiple lesions had high anti-rKRP42 IgG titers above the second quartile. The size, clinical form, or associated characteristics such as pruritus, scaling, etc., did not show any significant association with seropositivity (*p* > 0.05). All individuals (*n* = 11; 100.0%) with chronic lesions (>12 months) were seropositive.

## 4. Discussion

Physicians, especially dermatologists, are encountering many diagnostic dilemmas in the confirmation of CL and atypical presentations are the most challenging entity. To overcome these diagnostic challenges in Sri Lanka, clinicians have deployed the PCR method, which is invasive, expensive, sophisticated, and not freely available. In our study, the commonly available diagnostic test, SSS, failed to diagnose over 20% of CL cases and up to 70% of cases with atypical lesions (Table 4). Therefore, we propose the combination of the routine diagnostic method with serum ELISA to overcome said limitations. 

ITS1 PCR is a genus-specific assay that has been successfully used for the identification of *Leishmania* parasites from clinical lesions [28,29], especially for the detection of Old World species [30]. Since ITS1 PCR is already validated for CL in Sri Lanka [31] and the novel primers would only enhance its sensitivity and specificity, the nested ITS1 PCR was used as the gold standard in this study. 

The combination of SSS (technician) and serum ELISA was almost 99% sensitive for case detection against the gold standard—nested PCR. A fair and significant agreement between combination and PCR was observed (Kappa = 0.378; *p* < 0.01). Though the PPV remained over 96%, NPV (60%) and specificity (30%) were lower. However, the high PPV of the combination method (>96%), would minimize false positives, and NPV was also more favorable at 60%. Relatively low NPV and very low specificity may be due to inbuilt issues (discussed in the next paragraphs) associated with antibody-based diagnostics. However, the combination has detected more true cases and performed exceedingly well in the clinically doubtful category (detecting 16/17 cases equal to nested PCR where SSS alone was not efficient (94.1% vs. 29.4%; *p* = 0.0001)). Most CL patients who come to dermatology clinics in Sri Lanka, even at peripheral units, are routinely investigated with SSS and full blood count. Therefore, conducting serum-based ELISA will not add risk to patients. The infection risk of the combination test (need superficial skin slit and a sample of intravenous blood) should compare favorably to the PCR method (requiring a punch biopsy of the lesion). The combination test would cost-effectively complement the clinical diagnosis and be carried out in resource-poor settings. Moreover, our data highlighted the value of the combination of SSS and serum ELISA at a time when a rising trend of clinically doubtful atypical presentations is seen (Table 4).

The serum ELISA alone could detect a higher percentage (94.4%) of confirmed CL cases as seropositive. This is the highest reported seroprevalence by an ELISA-based method in Sri Lanka to date. The high sensitivity of the serum-based ELISA (94.4%) and high PPV (97.1%) favor the test as a complementary diagnostic test. Such high rates are unusual for CL, which is localized to the skin where disease resolution mainly depends on Th1 dominant cell-mediated response [32]. One study in Lebanon reported a high detection rate of parasites in blood in people with isolated cases of CL [33] which may favor a higher humoral response. Although this phenomenon has not been directly proven for the strain in Sri Lanka, the possibility exists due to the visceralizing potential of the Sri Lankan strain and might contribute to the high seroprevalence. This remains speculative, however, as visceralization following cutaneous disease has not been established in murine models [34]; hence, more studies are warranted. 

Titers of IgG and its subclasses are well documented to be correlated with active CL and VL and its clinical cure [34]. IgG1, IgG2, and IgG3 were shown to be upregulated with a dominant Th1-like CD4+ response in patients with active CL [35], and these subclasses were higher among CL than non-CL [36]. The present study has demonstrated higher total IgG titers in active CL compared to non-CL. This supports the value of anti-*Leishmania* IgG as a diagnostic marker to evaluate active CL. Serological or urine-based assays additionally offer the advantages of minimally/non-invasive sample collection and less labor-intensive analysis of samples in large batches and they are better suited (especially urine-based assays) for large-scale epidemiological studies. In our experience, people more willingly provided a sample of urine/blood than a tissue biopsy or skin slit smear.

Seropositivity rates and antibody titers were higher among people with multiple lesions (100.0%) compared to those with single lesions (90.2%; *p* < 0.05). A similar finding was observed among patients with multiple CL lesions caused by *L. tropica* (Wright, 1903) [37,38], and anti-*Leishmania* IgG2 was shown to be directly correlated to the number of lesions in patients with diffuse cutaneous leishmaniasis caused by *L. braziliensis* (Vianna, 1911) [39,40]. Multiple lesions could be due to multiple inoculating bites by the vector. The parasite burden in a person may therefore be higher due to lymphatic or dermal transmission via inflammatory monocytes [41]. Consequently, a higher antigenic stimulation of the immune system may lead to a higher immune response in an attempt to eliminate a higher burden of parasites. Alternatively, higher IgG might themselves contribute to multiple lesions. In VL, it was demonstrated that high IgG titers may contribute to disease progression by forming immune complexes that induce activated macrophages to secrete high IL-10, a potent inhibitor of macrophage activation that leads to parasite survival [42]. Several studies have shown a positive correlation between immune response producing activated T cells and inflammatory cytokines such as interferon-gamma and lesion size [43,44]. Larger lesions could secrete more cytokines and may stimulate higher production of IgG [36,43]. This association was not observed in the present study. 

Some confirmed CL cases (*n* = 10) were missed by serum ELISA, probably owing to the lower humoral response in this subset of patients, the detection of which was likely hindered by the sensitivity of the described ELISA. Individual variations in antibody production, age [45], and immune status, including transient immune suppression states, may have contributed. Only a fair (but significant) correlation was observed between the paired antibody titers in serum and urine among the CL cohort (r = 0.221; *p* < 0.01). This probably occurred due to lower urine antibody titers in CL, the detection of which may have been further hindered by the lower sensitivity of the urine-based ELISA. Contrastingly, in VL, both serum and urine-based ELISAs have shown comparable sensitivity [24]. In the present study, both serum and urine-based ELISAs demonstrated low specificity (50%) compared to nested PCR, mainly because both serum and urine-based ELISAs detected several positives among PCR (reference standard) negative cases. The sensitivity of parasitological techniques, including PCR, may vary depending on the parasite burden in the collected sample. This is impacted by sampling site, lesion duration, host immunity [46,47], the expertise in sample collection, processing, and PCR-related factors. Effects are more pronounced at ultra-low parasite densities especially because parasites tend to be clustered [47]. However, exposure to the parasite can be detected with a sufficiently sensitive serological test even when the above factors may hamper the sensitivity of parasitological tests. Despite the many advantages, antibody detection tests come with the limitation of low specificity in endemic areas due to early, subclinical, and past infections or cross-reactions, thereby reducing their standalone value as diagnostic tests. However, this study supports the value of serology, especially when clinical diagnosis is hampered by atypical variants. 

The presence of different *Leishmania* species or subspecies in a region may also influence the sensitivity and specificity of antibody-based tests. However, this factor probably played a minor role in the present study for the following reasons. (i) No species other than *L. donovani* has been reported from Sri Lanka to date. (ii) Restriction fragment length polymorphism (RFLP) testing of all the samples from our study confirmed that the only causative species among the study cohort was *L. donovani* (data not shown). (iii) The rKRP42 antigen test has high sensitivity and specificity to detect *L. donovani* [24]. However, the presence of subspecies, host genetics, and other factors influencing immunity may still account for differences in test performance.

In the present study, we have analyzed the response to rKRP42 and rK39 using different techniques, viz., ELISA and immunochromatographic test (ICT) respectively which tends to make their comparison difficult and it would have been optimal to employ the same technique. However, earlier studies that compared the performance of ELISA and ICT for rK39 have demonstrated that both performed almost equally in the diagnosis of VL confirming that the accuracy of the ICT for the diagnosis is as good as ELISA [48]. At the same time, rK39 antigen has shown wide variability in its performance among different geographic populations with VL [24] while its positivity was consistently low among Sri Lankan CL patients [20,22]. Based on these facts we decided to explore the performance of a different antigen for the diagnosis of CL in Sri Lanka.

The consistently low rK39 antibody detection among CL in Sri Lanka is probably an indication of the differences in the antigenic composition of the local strain [20]. This is supported by the fact that VL cases in Sri Lanka have not shown a consistent rK39 antibody response [20]. Interestingly, our study reports a 9% rate, the highest rate reported in a Sri Lankan study, for rK39 positivity, which was significantly associated with having very high titers of anti-rKRP42 IgG (unit value >251.3; *p* < 0.01). In co-endemic areas of VL and CL (India and citizens of the United States of America with CL), a small but variable percentage of CL patients have been shown to give positive results for the rK39 assay attributed to cross-reactivity, subclinical infections, or higher parasitic burden in multiple and large lesions [49,50]. In the present study, the majority were in single, early, and small lesions. Early lesions harbor higher parasite densities, supporting the assumption of a higher parasitic burden [46,51]. However, viseralization and parasitemia are possibilities [10,33]. Hence, they need to be followed up, especially because VL runs an indolent course and common asymptomatic and subclinical infections. Furthermore, cross-reactions and consequent false positives are known with malaria, tuberculosis, leprosy, trypanosomiasis, and Chagas disease [49]. None of the subjects had clinical evidence of any of these infections. However, the possibility of having subclinical infections is unlikely because there is no indigenous transmission of malaria in Sri Lanka. Moreover, trypanosomiasis or Chagas disease is not reported in Sri Lanka. Hence laboratory tests were not used to exclude these infections in the patient cohort. A study conducted in Bangladesh showed that high anti-rKRP42 urine ELISA titers of >1000 units could help predict VL cases [52]. The titers of anti-rKRP42 ELISA varied between 28 and 2423 units in serum (cutoff = 27.5 units) and 7 and 810 units in urine (cutoff = 6.5 units) in the CL cases we studied. Although the titers are not as high as in VL, many local CL cases have had serum antibody titers well over 1000 units. This is reinforced by the association of rK39 positivity with high anti-rKRP42 ELISA titers and highlights the need for long-term follow-up of such patients. Differences in the response to different antigens may also be due to the genetic diversity of *L. donovani*. Recent studies on genome analysis of the *L. donovani* complex showed vast diversity among *L. donovani* isolates which fell into five groups mainly based on geography while *L. infantum* showed little diversity [53]. Phylogenetic analysis of the Sri Lankan strains demonstrated that the genome of some strains had polymorphisms that are homologous with *L. tropica* (Wright, 1903) and *L. major* (Yakimoff and Schokhor, 1914). These hybrid strains probably originated from Africa and were phylogenetically distinct from Indian *L. donovani* strains [54]. Hybridizations, genome, and strain variations may contribute to the observed differences in our study.

It is important to discuss the present study’s challenges in developing cutoff value for the newly proposed ELISA. The main limitation of the present study was the unavailability of serum samples from healthy local controls from endemic and non-endemic areas in Sri Lanka for cutoff development. Japanese serum samples were used in place. Due to the difficulty in obtaining invasive samples from healthy controls, only urine samples were collected from the endemic control group. Therefore, local serum analysis was limited to the cohort of CL cases. However, collecting a non-endemic sample from Sri Lanka has its limitations because the disease is endemic to the entire island at present. Specificity assays for rKRP42 ELISA from other coexisting infectious diseases, including trypanosomiasis, leprosy, tuberculosis, etc., could not be performed due to the unavailability of biological samples. As expected, none were positive by serum ELISA in the JC group (*n* = 80) (Figure 3). Although urine rKRP42 ELISA has been successfully used for VL [24], it was suboptimal for the diagnostic purposes in CL. However, it detected over 60% of the CL cases and has the potential to be optimized and used for epidemiological studies.

In the present study, there was no gender or age-related difference in seroconversion rates. Both genders with a slight male preponderance (female = 90.7%; male = 93.0%) and all age groups showed almost equal seroconversion rates. Similarly, occupational exposure or the low-income category had no significant impact on seroconversion. These findings are aligned with the findings on smear and nested PCR positivity and suggest peri-domestication of the transmission cycle with indiscriminate impact on all categories of people. A previous study on seroprevalence in Sri Lanka has shown a higher seroconversion rate among females, small lesions, and a lower rate of seroconversion among the elderly [20]. A study on VL in Iran also demonstrated rapidly decreasing seroconversion rates with increasing age [45]. However, in our study, people over 61 years of age showed an almost equal seroconversion rate to other age groups. It may be difficult to observe these differences in a cohort of CL cases, and a large community-based survey similar to the Iranian study would provide more interpretable results [45].

The southern focus of CL has continued to expand over the years [25]. Control of leishmaniasis depends on case detection and treatment. Expertise and experience with both clinical and laboratory diagnosis have evolved over the years, but considerable gaps remain. As mentioned above, the clinical diagnosis followed by SSS is the first-line screening tool used in dermatology clinics. The majority of the lesions in this study could be diagnosed on clinical grounds as classical CL (*n* = 173). A minority (*n* = 17), such as acne-like small papules, macular lesions, and large ulcers with irregular edges were identified clinically as doubtful cases. Clinical features of CL are not pathognomonic, and conditions such as cutaneous tuberculosis, leprosy, fungal infections, and some non-infectious skin conditions like lupus, squamous cell carcinoma, and psoriasis may also resemble different types of lesions associated with CL [55]. Hence, the value of laboratory diagnosis remains undeniable before committing a patient to a long course of potentially toxic treatment. The utility of SSS remains high in the local setup due to its low cost and availability despite the variable sensitivity. This study revealed that the SSS at a sensitivity of 76.3% (technicians, routine CL diagnosis) in a government hospital is missing over 20% of the cases. Therefore, the proposed approach may reduce the burden of a national campaign to combat with rising cases of CL.

## Figures and Tables

**Table 1:** Clinical and demographic characteristics of the CL cohort (*n* = 190).

Variable		Number (%)
Clinical diagnosis	Confirmed	173 (91.0)
	Doubtful	17 (9.0)
Gender	Female	75 (39.0)
	Male	115 (61.0)
Age	<40 years	73 (38.4)
	41–60 years	87 (45.8)
	>61 years	30 (15.8)
Occupation	Occupied	132 (69.5)
	Unoccupied	58 (30.5)
Income (LKR))	<30,000	92 (48.4)
	30,000–50,000	76 (40.0)
	50,000–100,000	16 (8.4)
	>100,000	6 (3.2)
Lesion duration	< = 4 months	127 (67.0)
	4–12 months	52 (27.4)
	>12 months	11 (5.8)
Number of lesions	Single	153 (80.5)
	Multiple	37 (19.5)
Size	<2 cm	165 (86.8)
	>2 cm	25 (13.2)
Nature of the lesion	Papule	23 (12.1)
	Nodule	32 (16.8)
	Ulcerated nodule	76 (40.0)
	Dry ulcer	26 (13.7)
	Wet ulcer	14 (7.4)
	Plaque	19 (10.0)
Site	On limbs	163 (85.8)
	Elsewhere	27 (14.2)

**Table 2:** Anti-rKRP42 IgG seropositive rates among CL cases and Japanese controls.

	Clinical CL	Confirmed CL	Japanese Controls
Total	190	180	80
Positives	175	170	0
Positive %	92.1	94.4	0.0

**Table 3:** Anti-rKRP42 IgG titers in urine.

	Clinical CL	Confirmed CL	Endemic Controls	Japanese Controls
Total	190	180	255	80
Positives	116	111	83	3
Positive %	61.1	61.7	32.5	3.75

**Table 4:** Comparison of the diagnosis of clinically doubtful lesions (*n* = 17) by different techniques.

Test	Positive *n* (%)	Negative *n* (%)	*p*-Value ^¥^	Sn (PPV) ^δ^	Sp (NPV) ^δ^
SSS (technician)	5 (29.4)	12 (70.6)	1.000	25% (80%)	0% (0%)
SSS (authors)	10 (58.8)	7 (41.2)	0.083	62.5% (100%)	100% (14.3%)
PCR	16 (94.1)	1 (5.9)	0.0001 *	-	-
rKRP42 serum ELISA	15 (88.2)	2 (11.8)	0.0006 *	93.8% (100%)	100% (50%)
Combination of SSS ^₶^ and rKRP42 Serum ELISA	16 (94.1)	1 (5.9)	0.0001 *	100% (100%)	100% (100%)

Sn—sensitivity; Sp—specificity; PPV and NPP—positive and negative predictive values, ^₶^ SSS by technicians, ^¥^ significance of sensitivity deference was calculated against SSS by technicians, * *p* < 0.001, ^δ^ calculated considering PCR as the gold standard.

## Data Availability

Data are contained within the article.
